# Supplementary material for: 5-HT2AR and NMDAR psychedelics induce similar hyper-synchronous states in the rat cognitive-limbic cortex-basal ganglia system
Source: Commun Biol. 2023 Jul 26;6:737. doi: 10.1038/s42003-023-05093-6 (PMC10372079; doi:10.1038/s42003-023-05093-6)

SUPPLEMENTARY INFORMATION

for

5-HT2AR and NMDAR psychedelics induce similar hyper-synchronous states in the rat cognitive-limbic cortico-basal ganglia system

Ivani Brys, Sebastian Barrientos, Jon Ezra Ward, Jonathan Wallander, Per Petersson, Pär Halje

Corresponding author: Pär Halje
E-mail: par.halje@med.lu.se

This PDF includes:
- Supplementary Tables S1-S7
- Supplementary Figures S1-S12

Table S1: Electrodes grouped on anatomical location

Table listing all electrodes grouped on their anatomical location according to the atlas (Paxinos & Watson, 2007). The anatomical locations were further grouped into 10 functional groups. The location of the wires in the "Wires (CT)" column were validated using computer tomography, while the location of wires in the "Wires (no CT)" were inferred from stereotaxic coordinates.

Abbriviation	Anatomical name	Functional group	Group abbreviation	Wires (CT)	Wires (no CT)
AOE	anterior olfactory nucleus, external part	Olfactory cortex	OC	1	
AOL	anterior olfactory nucleus, lateral part	Olfactory cortex		4	
AOP	anterior olfactory nucleus, posterior part	Olfactory cortex		13	
DEn	dorsal endopiriform nucleus	Olfactory cortex		8	
IEn	intermediate endopiriform nucleus	Olfactory cortex		1	
EPl	external plexiform layer of the olfactory bulb	Olfactory cortex		2	
Gl	glomerular layer of the olfactory bulb	Olfactory cortex		5	
GrO	granular cell layer of the olfactory bulb	Olfactory cortex		2	
Mi	mitral cell layer of the olfactory bulb	Olfactory cortex		2	
PLCo	posterolateral cortical amygdaloid nucleus	Olfactory cortex		1	
Pir	piriform cortex	Olfactory cortex		20	
AIV	agranular insular cortex, ventral part (bordering LO)	Orbitofrontal cortex	OFC	3	
DLO	dorsolateral orbital cortex	Orbitofrontal cortex		4	
LO	lateral orbital cortex	Orbitofrontal cortex		31	18
MO	medial orbital cortex	Orbitofrontal cortex		15	
VO	ventral orbital cortex	Orbitofrontal cortex		15	
FrA	frontal association cortex	Prefrontal cortex	mPFC	8	
IL	infralimbic cortex	Prefrontal cortex		1	12
PrL	prelimbic cortex	Prefrontal cortex		36	62
Au2	secondary auditory cortex	Temporal association area	TAA	4	
Ect	ectorhinal cortex	Temporal association area		5	
DLEnt	dorsolateral entorhinal cortex	Temporal association area		1	
HippD	dorsal hippocampus	Temporal association area			16
HippV	ventral hippocampus	Temporal association area			94
MoDG	molecular layer of the dentate gyrus	Temporal association area		8	
Or	oriens layer of the hippocampus	Temporal association area		9	
opt	olivary pretectal nucleus (bordering hippocampus)	Temporal association area		1	
M1	primary motor cortex	Sensorimotor cortex	SMC	11	
M2	secondary motor cortex	Sensorimotor cortex		20	12
S1	primary somatosensory cortex	Sensorimotor cortex		58	
S2	secondary somatosensory cortex	Sensorimotor cortex		1	
AcbC	accumbens nucleus, core	Ventral striatum	vStr	1	42
AcbSh	accumbens nucleus, shell	Ventral striatum		8	30
CPuV	ventral striatum	Ventral striatum		1	
Tu	olfactory tubercle	Ventral striatum		10	
CPuDL	dorsolateral striatum	Dorsal striatum	dStr	15	8
CPuL	lateral striatum	Dorsal striatum		2	
CM	central medial thalamic nucleus	Integrative thalamus	IntThal	1	
LDDM	laterodorsal thalamic nucleus, dorsomedial part	Integrative thalamus		1	
MD	mediodorsal thalamic nucleus	Integrative thalamus		6	79
PT	paratenial thalamic nucleus	Integrative thalamus		2	
AD	anterodorsal thalamic nucleus	Associative thalamus	AssocThal	2	
AM	anteromedial thalamic nucleus	Associative thalamus		15	
AV	anteroventral thalamic nucleus	Associative thalamus		13	
MG	medial geniculate nucleus	Sensory thalamus	SensThal		42
Po	posterior thalamic nuclear group	Sensory thalamus		1	
VG	ventral geniculate nucleus	Sensory thalamus		2	
VPL	ventral posterolateral thalamic nucleus	Sensory thalamus		9	
VPM	ventral posteromedial thalamic nucleus	Sensory thalamus		2	
Rt	reticular thalamic nucleus	Sensory thalamus		1	

Table S2: Number of cells grouped on functional group and drug

Number of cells

Location	5HT2A	NMDA	Amphetamine
Associative thalamus	3	2	0
Integrative thalamus	13	20	6
Olfactory cortex	7	4	0
Orbital cortex	17	12	2
Prefrontal cortex	18	19	18
Sensorimotor cortex	5	6	1
Dorsal striatum	2	2	0
Sensory thalamus	1	22	2
Temporal association area	40	38	9
Ventral striatum	33	38	25

Drug

Table S3: Entrainment strength on psychedelics (mean kappa±SEM)

Structure	PC	IN	X
Olfactory cortex	0.09 ± 0.04		0.05 ± 0.01
Orbitofrontal cortex	0.10 ± 0.02	0.04 ± 0.01	0.03 ± 0.01
Medial prefrontal cortex	0.15 ± 0.06	0.07 ± 0.02	0.08 ± 0.02
Temporal association area	0.24 ± 0.04	0.09 ± 0.02	0.17 ± 0.03
Sensorimotor cortex	0.06 ± 0.03	0.03 ± 0.00	
Ventral striatum	0.27 ± 0.06	0.03 ± 0.01	0.24 ± 0.02
Integrative thalamus			0.11 ± 0.02

Table S4: Electrodes grouped on functional group and animal

Table showing the number of wires in each functional group split on animals. Animals ER, ES, FA, FB and FC were CT scanned.

Number of wires

Location	EA	EJ	EM	ER	ES	FA	FB	FC	FD
Olfactory cortex	0	0	0	8	2	0	23	26	0
Orbital cortex	0	0	0	6	10	12	21	19	18
Prefrontal cortex	19	17	14	8	0	25	12	0	24
Temporal association area	23	39	20	0	15	12	1	0	28
Sensorimotor cortex	0	0	0	0	1	33	31	25	12
Ventral striatum	22	21	17	5	9	0	3	3	12
Dorsal striatum	0	0	0	0	1	3	11	2	8
Integrative thalamus	23	24	16	0	2	1	6	1	16
Associative thalamus	0	0	0	14	7	1	6	2	0
Sensory thalamus	10	16	16	8	6	0	1	0	0

Animal

Table S5: List of experiments

Animal	Day	Treatment	Dose mg/kg
EA	0	ket	50
EA	2	ket	25
EA	6	ket	50
EA	9	ket	50
EA	13	LSD	0.3
EA	15	LSD	0.3
EA	27	amphet	4
EA	31	amphet	4
EA	49	ket	50
EA	76	LSD	0.3
EA	87	ket	50
EA	99	ket	50
EA	106	ket	50
EA	112	LSD	0.3
EJ	0	ket	50
EJ	7	LSD	0.3
EJ	10	LSD	0.3
EJ	14	ket	50
EJ	17	PCP	5
EJ	23	PCP	5
EJ	28	ket	50
EJ	30	LSD	0.3
EJ	36	PCP	5
EJ	73	amphet	4
EJ	77	DOI	2
EJ	79	DOI	2

Animal	Day	Treatment	Dose mg/kg
EM	0	amphet	4
ES	0	LSD	0.3
ES	5	ket	50
ES	6	PCP	5
ES	7	DOI	2
ES	11	amphet	4
ES	12	LSD	0.3
ES	18	DOI	2
ER	0	DOI	2
ER	3	ket	50
ER	5	PCP	5
ER	6	LSD	0.3
ER	7	amphet	4
ER	11	DOI	2
ER	13	DOI	2
ER	14	PCP	5
FA	0	ket	50
FA	3	LSD	0.3
FA	7	amphet	4
FA	53	ket	25
FA	63	ket	25
FA	74	ket	25
FA	133	ket	25
FA	143	ket	25

Animal	Day	Treatment	Dose mg/kg
FB	0	LSD	0.3
FB	3	ket	25
FB	5	LSD	0.3
FB	7	ket	25
FB	9	amphet	4
FB	13	ket	25
FB	15	ket	25
FB	19	LSD	0.3
FB	23	DOI	2
FB	30	PCP	5
FC	0	LSD	0.3
FC	3	ket	25
FC	5	LSD	0.3
FC	7	ket	25
FC	9	amphet	4
FC	13	ket	25
FD	0	DOI	2
FD	7	LSD	0.3
FD	12	ket	25
FD	14	LSD	0.3
FD	17	amphet	4

Table S6: Behaviors assessed by scoring

Behavior	Definition
Being still	Being completely still with all paws touching the ground (cf "Lying down"). This presumably includes both wakeful resting and sleeping.
Grooming	Stereotyped rat self-grooming, including paw strokes on the head and body licking.
Rearing	Standing up on the hindlimbs.
Sniffing upwards	Sniffing and whisking with the head turned upwards.
Sniffing downwards	Sniffing and whisking with the head directed forward or toward the ground.
Head-swaying	Head swaying from side to side.
Moving backwards	Backward locomotion.
Intermittent turning	Short locomotion bouts that alternate left and right without resulting in any significant movement forwards.
Unstableness	Unstable posture or stumbling, wobbly gait.
Falling over	Severe unstableness resulting in falls while moving or standing still.
Lying down	Lying down on the side, unable to maintain a normal body posture. Often accompanied by limb movements.
Crawling	Movement of limbs with or without change of location while being unable to stand up

Table S7: Summary of spike shape features (mean±SEM µs)

Structure	Cell type	Peak width	Valley width	Peak-to-valley time
Olfactory cortex	PC	201 ± 25	368 ± 13	417 ± 11
	IN	122 ± 31	224 ± 31	215 ± 5
	X	175 ± 0	276 ± 0	325 ± 0
Neocortex	PC	243 ± 8	402 ± 8	506 ± 9
	IN	109 ± 3	252 ± 12	210 ± 9
	X	141 ± 6	406 ± 12	337 ± 7
Temporal association area	PC	194 ± 13	362 ± 11	433 ± 11
	IN	111 ± 4	211 ± 12	209 ± 10
	X	166 ± 12	276 ± 26	333 ± 9
Striatum	PC	266 ± 7	332 ± 7	427 ± 7
	IN	127 ± 6	234 ± 9	223 ± 15
	X	185 ± 15	254 ± 21	361 ± 13
Thalamus	X	128 ± 6	258 ± 17	289 ± 20

Figure S1: Manually scored behaviors

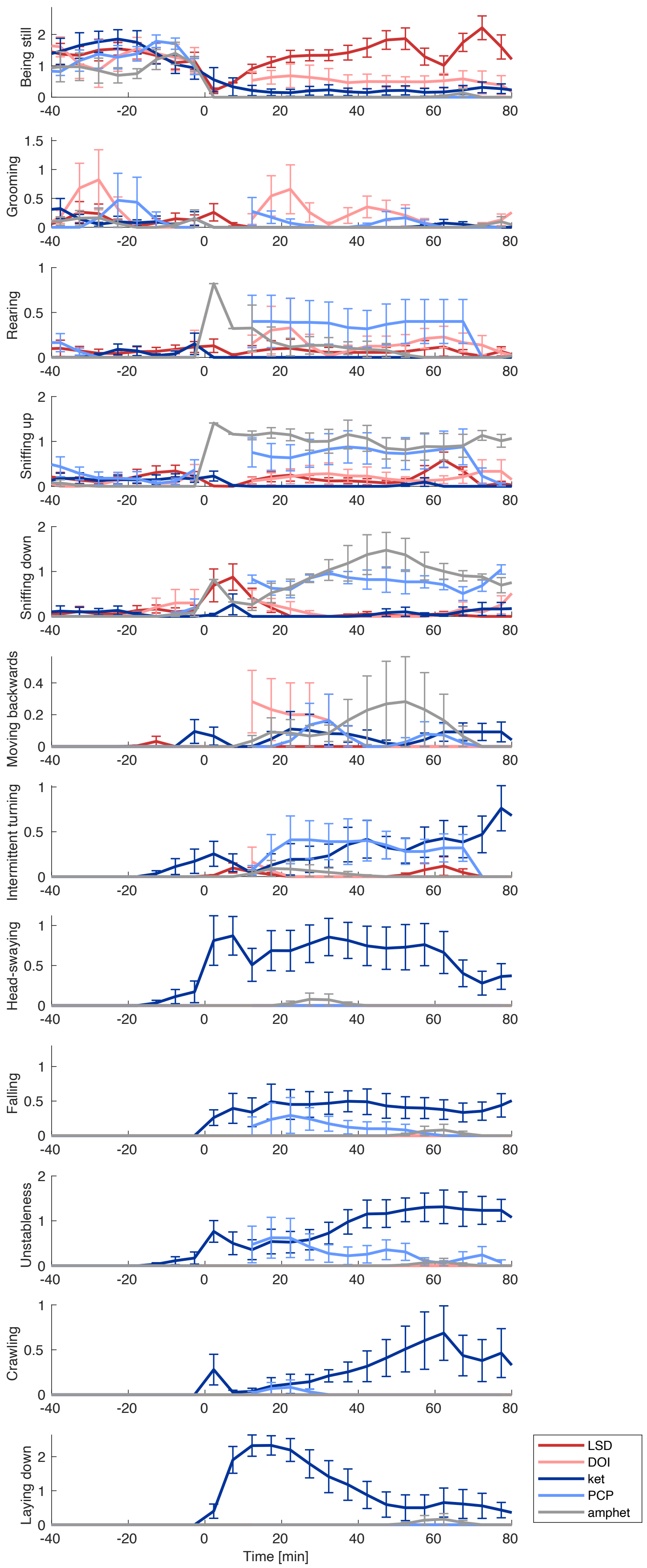

Time evolution of behaviors scored on a scale from 0 to 3 during 1 minute of observation each 10 minutes. Scores were interpolated in 5-minute steps and averaged across recording sessions. Lines show mean±SEM for LSD (red), DOI (pink), ketamine (dark blue), PCP (light blue) and amphetamine (grey). Times are relative to injection.

Figure S2: Summary of manually scored behaviors

Average changes in behavior for each condition (Base = baseline, 2A = LSD or DOI, NMDA = ketamine or PCP, Am = amphetamine), scored on a scale from 0 to 3. Data was averaged over the periods [-35 -5] minutes for baseline and [30 60] minutes for the other conditions (relative to injection). Bars show mean and SEM, asterisks show significance at the p<0.05 (*), p<0.01 (**) and p<0.001 (***) levels (nested ANOVA).

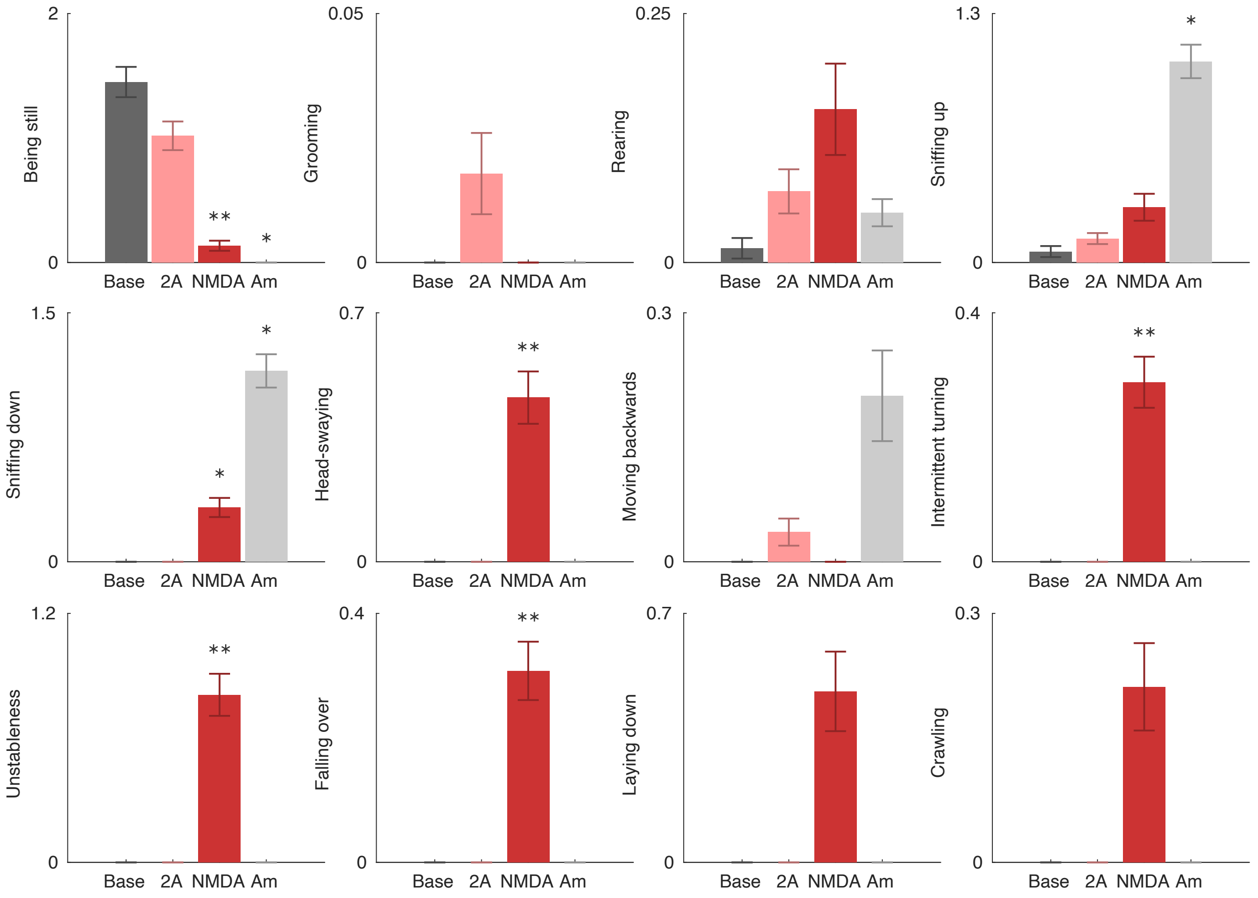

Figure S3: Firing rate responses to LSD, DOI, ketamine and PCP

Standardized neuronal firing rate responses to LSD, DOI, ketamine and PCP. Each row shows the activity of a single unit and rows are rank ordered according to the response during the drug period.

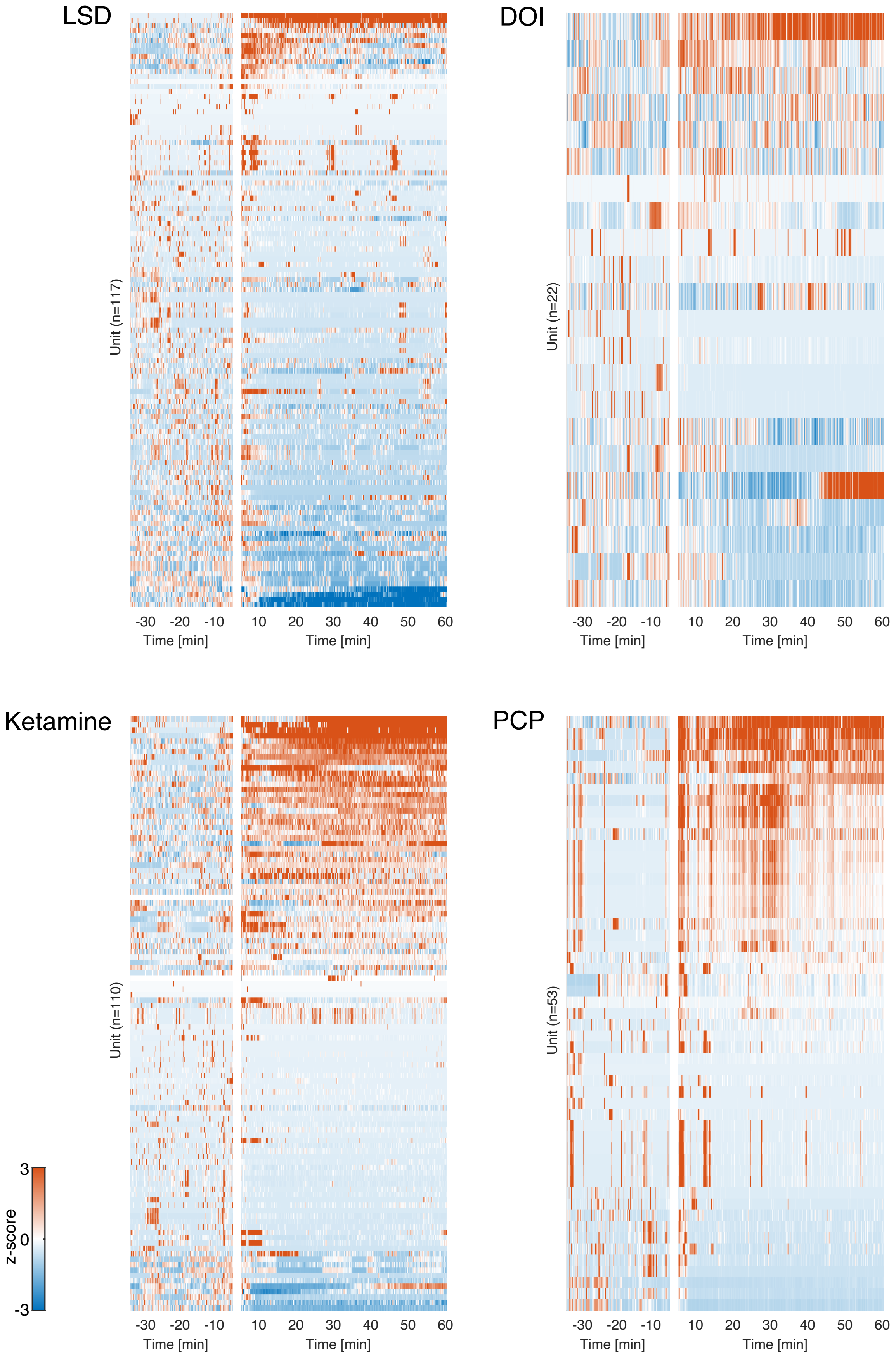

Figure S4: Classification of units into putative cell types

Scatter plots of waveform features (left column) and corresponding waveforms (right column). Three features were used in the classification: peak width (FWHM), peak-to-valley time and valley width (FWHM; not shown). Yellow = putative principal cell, blue = putative interneuron, grey = unclassified.

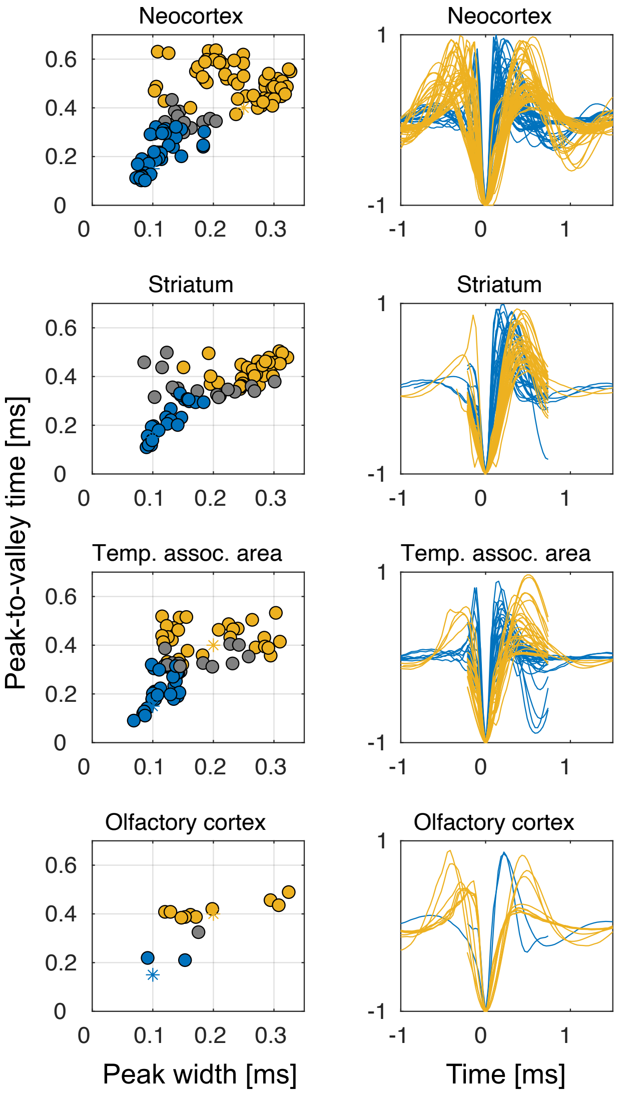

Figure S5: Firing rate modulations for LSD, DOI, ketamine and PCP

A. Average standardized neuronal firing rate responses to LSD, DOI, ketamine and PCP for different cell populations (PC=putative principal cells, IN=putative interneurons, X=unclassified cells). The response is calculated as average z-scores during 30 to 60 minutes post drug injection compared to baseline (-35 to -5 minutes). Asterisks indicate significance at the p<0.05 level (nested ANOVA). The numbers next to the cell labels indicate the number of cells in each population.
B. Fraction of modulated cells as response to LSD, DOI, ketamine and PCP for different cell populations (PC=putative principal cells, IN=putative interneurons, X=unclassified cells). The fractions of downmodulated cells are shown in blue and upmodulated cells are shown in red. Asterisks indicate significance at the p<0.05 level (binomial test).

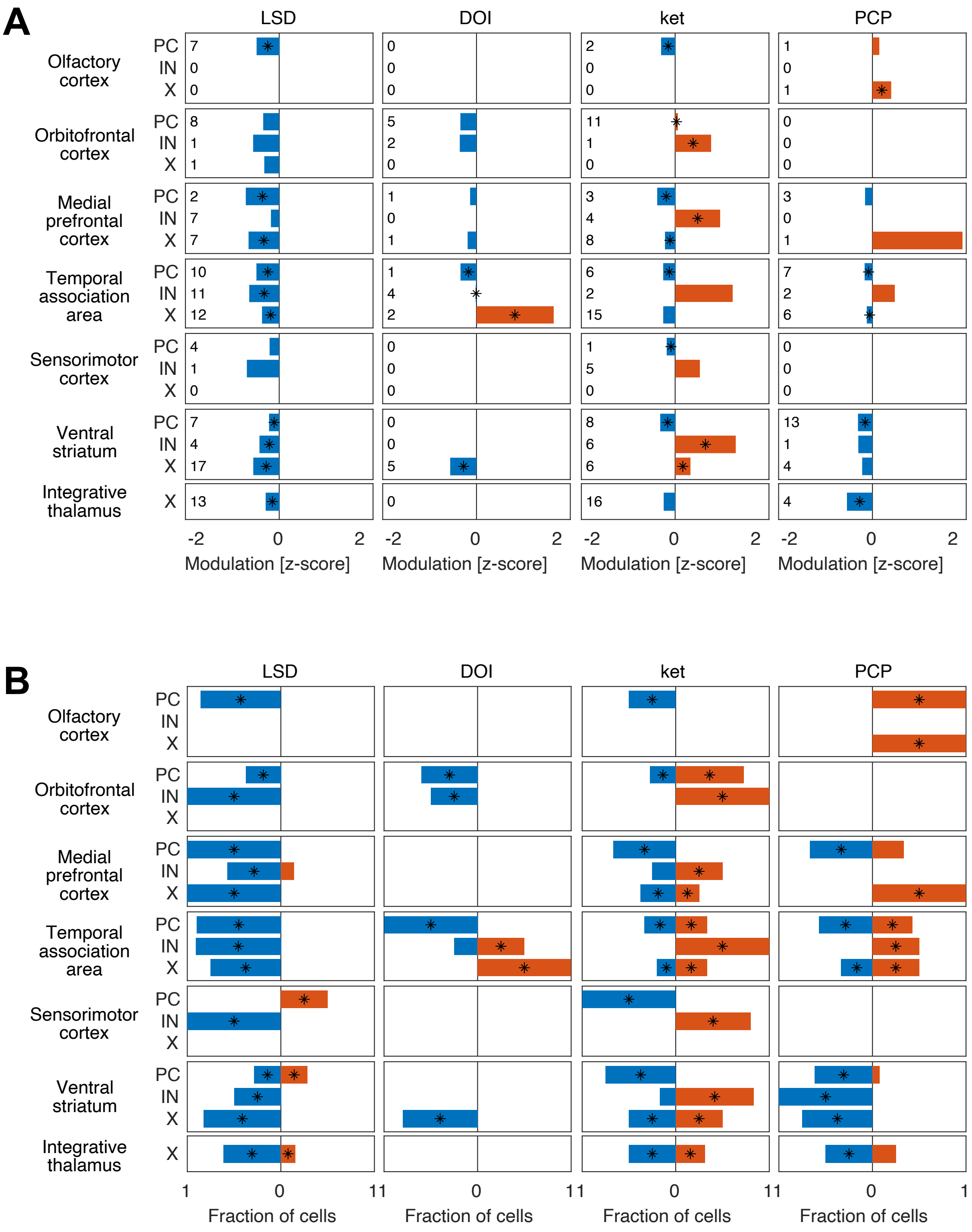

Figure S6: LFP power spectra in response to LSD, DOI, ketamine and PCP

LFP power spectra averaged over time and treatment groups (baseline=grey, drug=red). The time periods used were -35 to -5 minutes for baseline and 30 to 60 minutes for drug treatment relative to injection. Shaded areas show bootstrapped 95% confidence intervals.

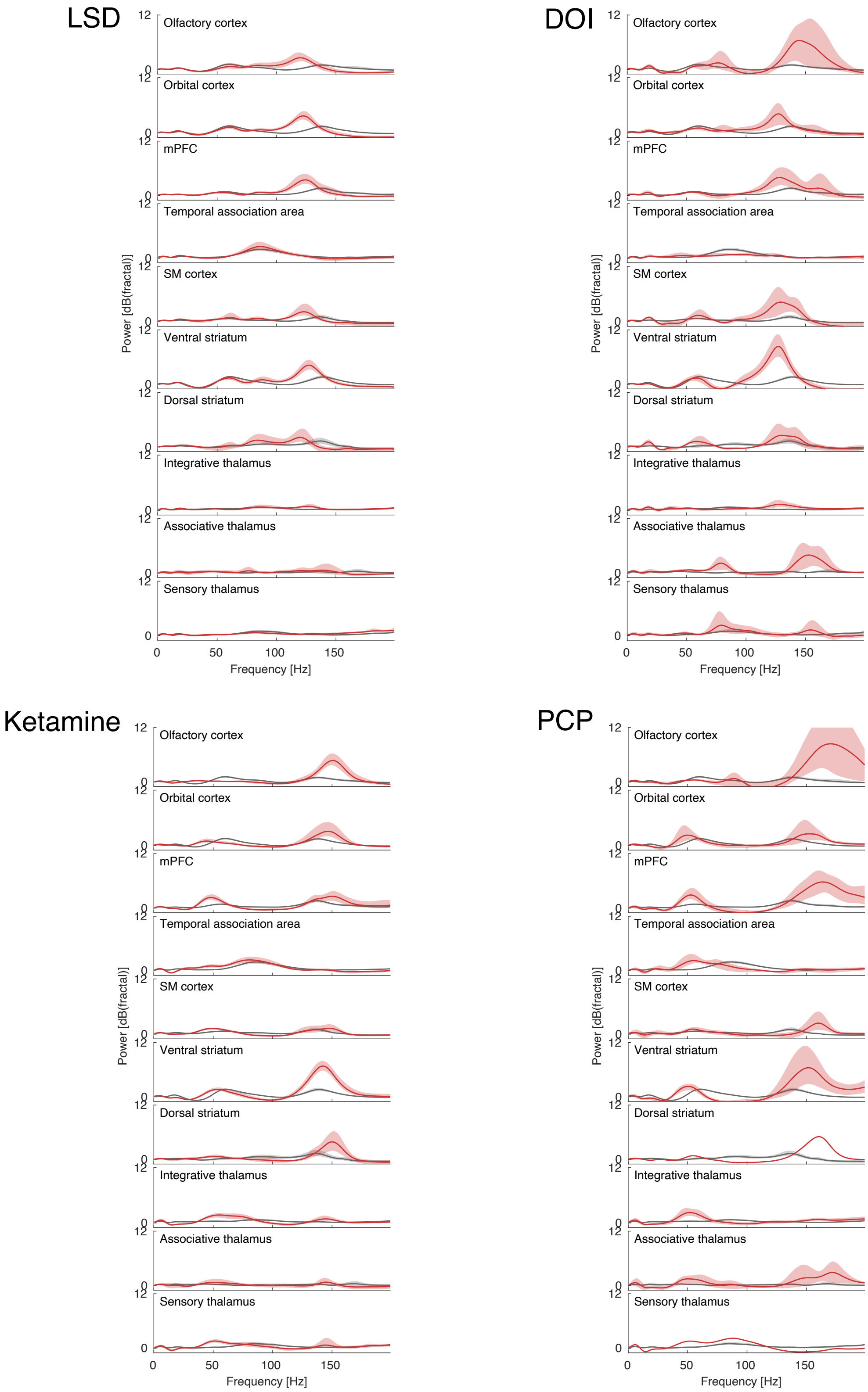

Figure S7: HFO detection

A. Examples of LFP power spectra (grey) from 8 seconds of data. Red or green lines show the corresponding fitted function

$$y(f) = a_1 e^{\left(\frac{f-a_2}{a_3}\right)^2} + a_4 + a_5.$$

Functions with parameter values and goodness-of-fit within given limits were counted as successful HFO detections and marked in green.
B. Spectrograms (same as in Figure 3B) with successful HFO detections marked with green crosses.

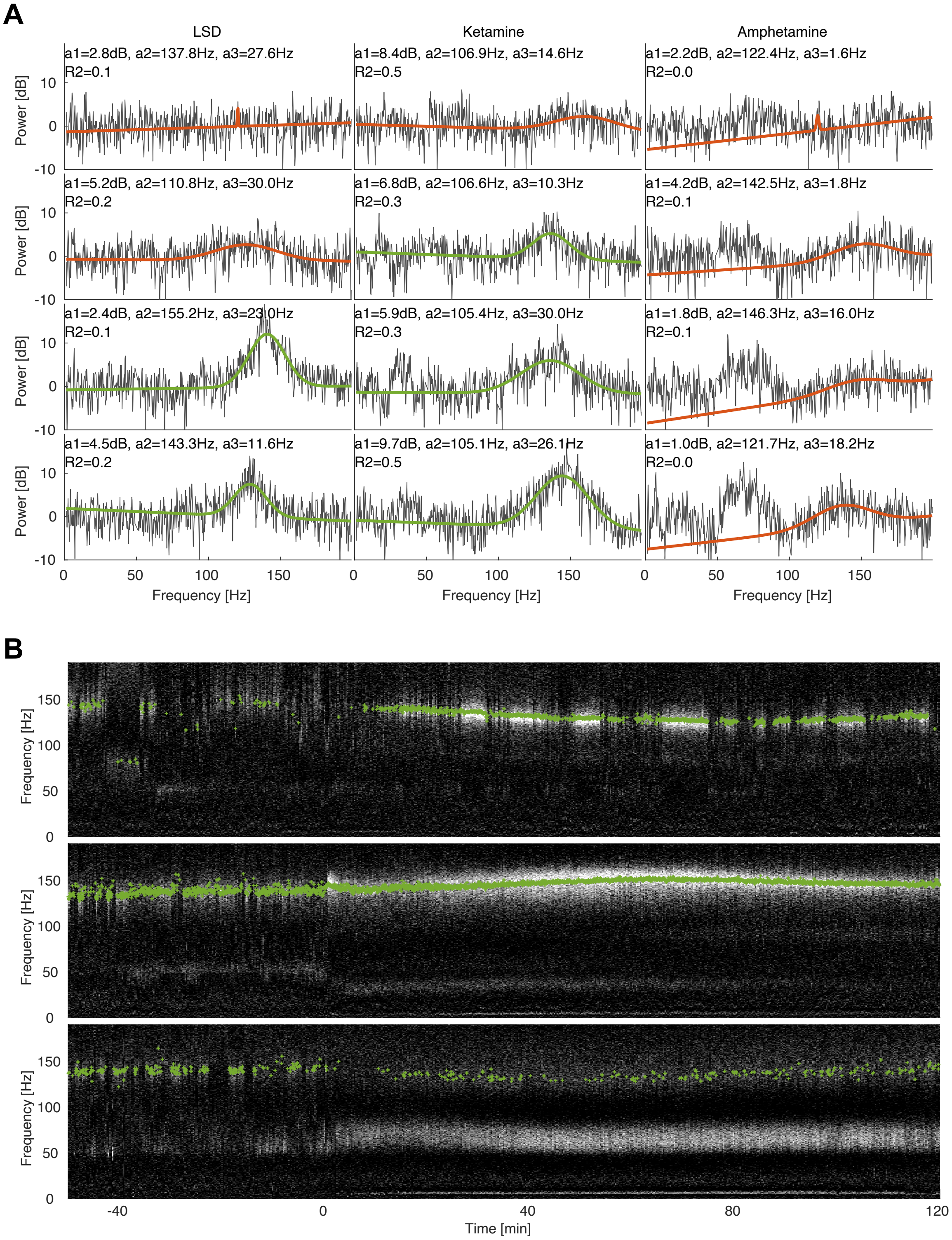

Figure S8: HFO detection rates

A. Time-course of HFO detection rates (mean=solid lines, SEM=dotted lines) aligned to the time of drug injection (dashed line). OC=olfactory cortex, OFC=orbitofrontal cortex, mPFC=medial prefrontal cortex, TAA=temporal association area, SMC=sensorimotor cortex, vStr=ventral striatum, dStr=dorsal striatum, IntTal=integrative thalamus, AssocThal=associative thalamus, SensThal=sensory thalamus.

B. Average HFO detection rates compared to baseline. Bars show mean and SEM. Asterisks indicate significance compared to baseline at the p<0.05 (*), p<0.01 (**) and p<0.001 (***) levels (nested ANOVA). Hash signs indicate significance compared to amphetamine. None of the pairwise comparisons between LSD, DOI, ketamine and PCP were significant.

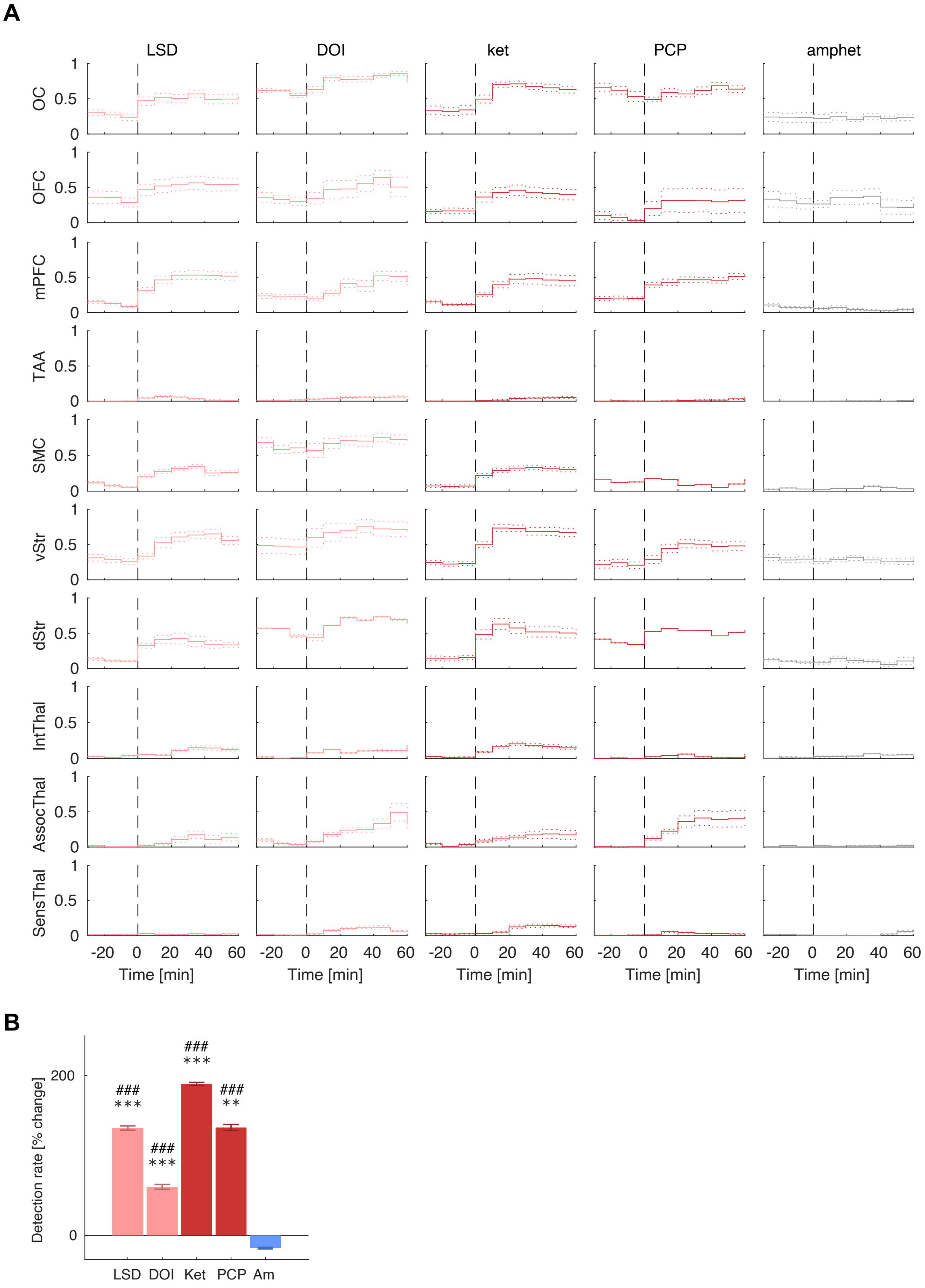

Figure S9: Classification of HFO detection rates

To better visualize how prevalent HFOs were in different structures and recording sessions, we defined 4 prevalence classes based on the distribution of detection rates. First, the detection rate was determined for each recording session and treatment state. The fraction of sessions with a higher rate than a given threshold was then plotted as a function of that threshold (pink lines = 5HT2A, red lines = NMDA, dark grey lines = baseline, light grey lines = amphetamine). HFOs were classified as being Persistent if the detection rate was higher than 90% in at least 33% of sessions (red zone). They were Prevalent if the detection rate was higher than 50% in at least 33% of sessions (orange zone). Otherwise HFOs were Occasional (yellow zone), unless the detection rate was higher than 5% in no more than 5% of sessions. Then they were classified as Absent (grey zone).

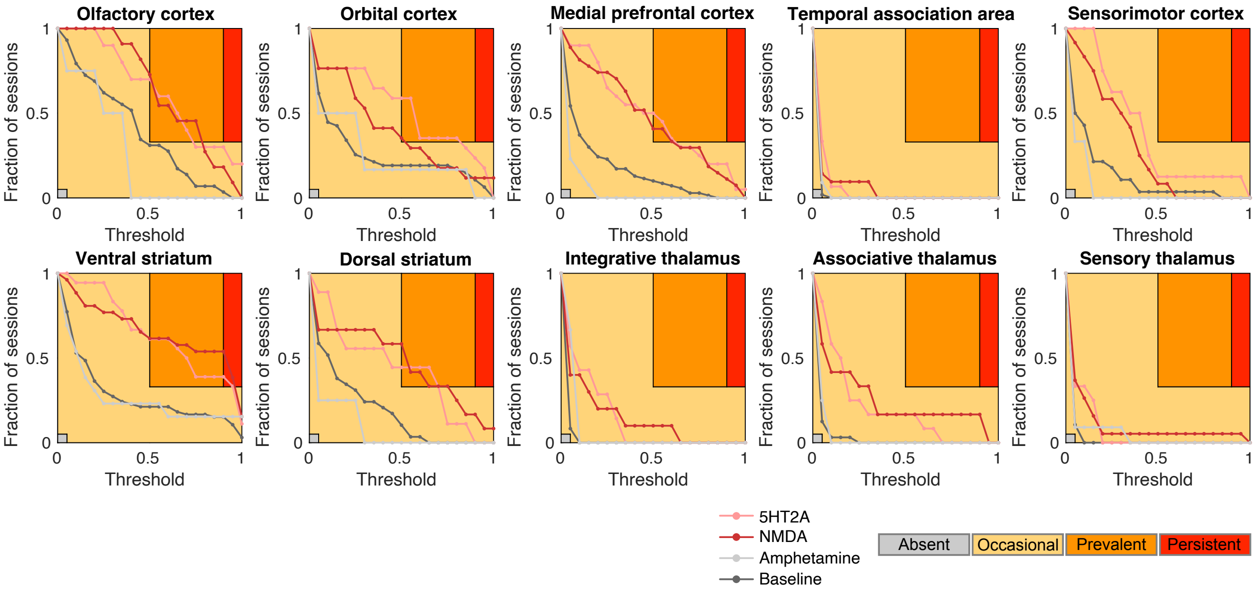

Figure S10: Simultaneous HFO frequencies in structure pairs during baseline

2D histogram showing the relationship between HFO frequencies in pairs of structures during baseline. Each data point comes from two simultaneously obtained spectra calculated from an 8 s time window.

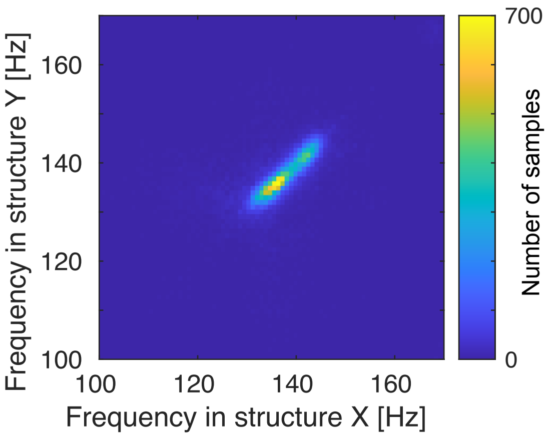

Figure S11: HFO phase-locking during baseline

Scatter plot showing mean phase differences and κ values for the phase difference distributions of each electrode pair during baseline. Most pairs had a non-random phase relationship (71% with κ>1). Dots are blue when $|\phi| < \pi/2$ and κ>1, and dots are red when $|\phi| > \pi/2$ and κ>1.

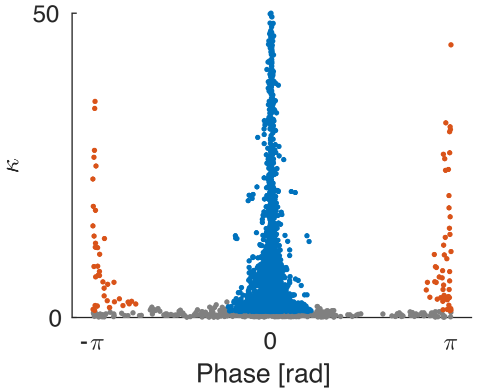

Figure S12: Validation of head-twitch response detections

A. The medial-lateral (ML) component of the acceleration shows a clear oscillation during the HTR event.
B. The spectrogram of the ML component shows that the oscillation is found between 8 and 32 Hz (magenta dashed lines).
C. Absolute value of C smoothed with a Gaussian window (σ=50 ms).

D. Example data from an LSD recording showing high correspondence between manual and automatic detection. Manually identified events were divided in three classes based on their intensity: 1 = short side-to-side movement of the head but not a clear shake; 2 = clear shake of the head and/or part of the anterior portion of the trunk; 3 = clear powerful shake with most of the body involved. Colored diamonds correspond to manually identified HTR events (blue = class 1, not shown; purple = class 2; red = class 3). Grey diamonds indicate HTR events detected by the accelerometer with a threshold at 0.4 (grey dashed line).
E. Distribution of HTR indices for manually detected HTR events, split on manually identified HTR classes (grey = non-HTR; blue = HTR1; purple = HTR2; red = HTR3). There is a clear separation between HTR and non-HTR events (grey distribution), and a partial separation between HTR classes 1-3.
F. ROC curves with True Positives defined as manual scores >= 1 (blue line), manual scores >=2 (purple line) or manual scores = 3 (red line). The area under the curve (AUC) shows a near perfect classification for all three cases.

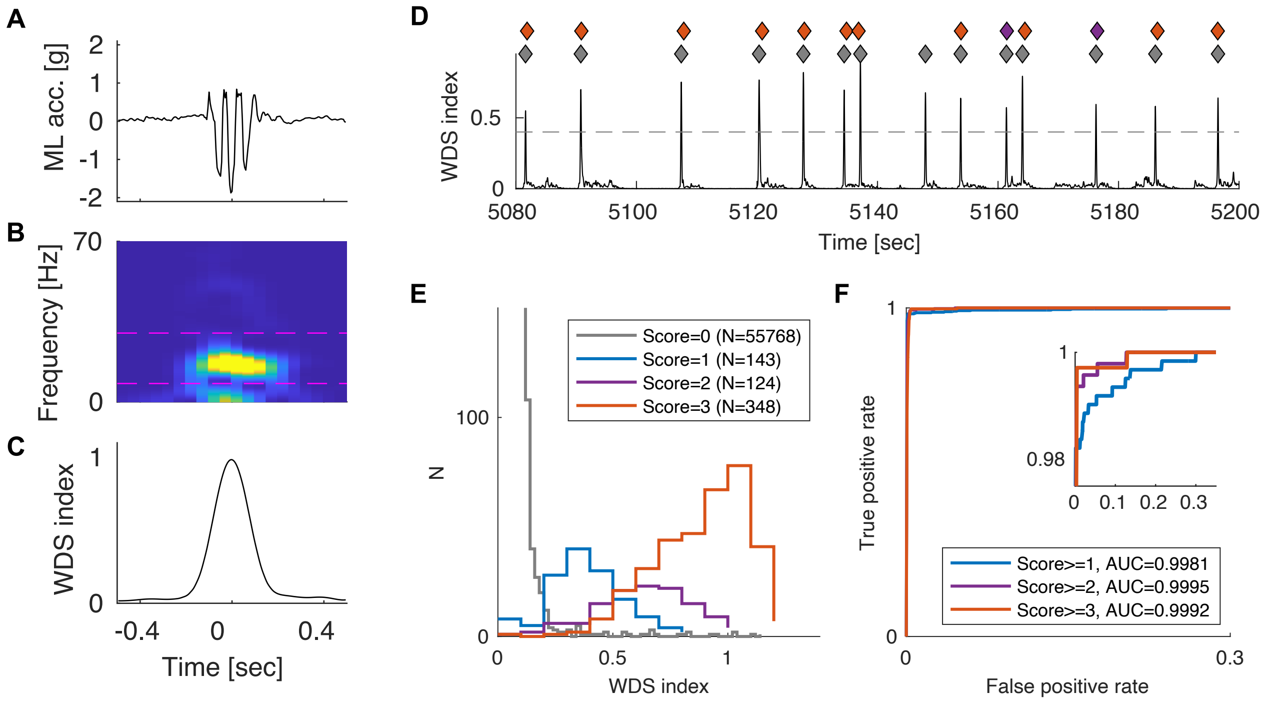

Supplement: Supplementary file 2 — Supplementary Information [file 42003_2023_5093_MOESM2_ESM.pdf]
